# Evaluating usability and acceptance of a socially assistive robot supported cognitive training for depression – results of the randomized controlled pilot study ‘AMIGA’

**DOI:** 10.3389/fpsyt.2025.1661730

**Published:** 2026-01-15

**Authors:** Alfred Haeussl, Ina Zwigl, Lena Stojec, Irina Smolak, Marko Stijic, Tatjana Stross, Melanie Lenger, Frederike T. Fellendorf, Suher Guggemos, Elena M. D. Schoenthaler, Martin Pszeida, Thomas Orgel, Sandra Draxler, Michael Schneeberger, Silvia Russegger, Julia Zuschnegg, Dominik Steindl, Anna Schultz, Sandra Schuessler, Michael Macher, Lucas Paletta, Nina Dalkner, Eva Z. Reininghaus

**Affiliations:** 1 Department of Psychiatry, Psychosomatic and Psychotherapy, Clinical Division of Psychiatry and Psychotherapeutic Medicine, Medical University of Graz, Graz, Austria; 2 Department of Healthcare and Nursing, Department of Health Sciences, Faculty of Health and Social Sciences, USTP – University of Applied Sciences St. Poelten, Sankt Poelten, Austria; 3 Department of Neurology, Division of Neurogeriatrics, Medical University of Graz, Graz, Austria; 4 DIGITAL – Institute for Digital Technologies, Joanneum Research Forschungsgesellschaft mbH, Graz, Austria; 5 Institute of Social Medicine and Epidemiology, Medical University of Graz, Graz, Austria; 6 Department of Media and Digital Technologies, Faculty of Engineering and Business, USTP – University of Applied Sciences St. Poelten, Sankt Poelten, Austria; 7 Humanizing Technologies GmbH, Vienna, Austria

**Keywords:** acceptance, usability, social assistive robotics (SAR), depression, cognitive training, sex differences, randomized controlled trial

## Abstract

The integration of socially assistive robots (SARs) in mental healthcare offers promising opportunities to enhance traditional treatments. The humanoid SAR “Pepper” has shown potential for supporting cognitive and emotional interventions for individuals. While SAR acceptance has been explored in general healthcare, limited evidence exists regarding its usability and acceptance among individuals with affective disorders. This study aimed to assess the usability and acceptance of “Pepper” as an adjunct motivational technology in combination with tablet-based cognitive training, compared to tablet training alone, among inpatients with depression, focusing on sex-specific differences. A randomized controlled trial was conducted between June and October 2024 with 32 inpatients diagnosed with depression. Participants were randomly assigned to one of two groups: the SAR group, which used “Pepper” together with the cognitive training app “Multimodal Activation” in combination with motivational feedback, and the control group, which used the same app on a tablet without motivational feedback. Each participant completed two cognitive training sessions of approximately 10 to 20 minutes. Standardised questionnaires, namely, *Technology Acceptance Model* (TAM), *System Usability Scale* (SUS), and *Technology Usage Inventory* (TUI), were administered after the second session. To analyze group and sex differences, analyses of co-variance were used for single-scale measures (SUS, TUI Overall), and multiple analyses of co-variances were used for instruments with multiple subscales (TAM, TUI). A significant group difference was found in the SUS score, favoring the SAR group. The SAR group also scored higher in the TUI *User-Friendliness*, while the tablet group showed higher scores in *Accessibility*. Regarding sex differences, female participants scored higher on the TUI *Overall* and *Scepticism* subscales than male participants. These findings suggest that SAR-supported cognitive training is a viable and accepted tool for supporting cognitive training in psychiatric care. High usability and positive acceptance ratings indicate its potential to complement conventional therapies. The observed sex-specific differences underline the relevance of tailored robotic interventions. Limitations of this study include the small sample size and short intervention period. Further studies are warranted to validate these findings and examine the ethical considerations and human–robot interaction dynamics in psychiatric settings.

## 1 Introduction

Depression is a widespread mental health disorder that affects over 264 million individuals worldwide (1). Treating depression often requires a multidimensional approach, including pharmacological treatment, psychotherapy/psychosocial interventions, and social support (2). In this context, socially assistive robots (SARs) could represent an innovative complement to traditional therapies and interventions. SARs are designed to interact with humans and assist them in various domains (3). The integration of robotic technology into healthcare has gained significant importance in recent years, particularly in mental health (4). SARs, such as the humanoid robot “Pepper”, offer promising opportunities to support patients with mental health conditions, particularly within clinical and therapeutic settings. By enhancing social interaction during therapy sessions, promoting cognitive stimulation through interactive exercises, and fostering emotional well-being in individuals with depression and anxiety disorders, SARs can contribute significantly to mental healthcare. These effects are facilitated by socially assistive behaviors, including empathetic verbal responses, adaptive nonverbal communication (e.g. gestures, gaze, or posture), and the ability to tailor interactions based on the user’s emotional state (5–7). However, the usability and acceptance of this technology among psychiatric patients, particularly those with depression, remain underexplored, and it is still unclear which patient groups are particularly responsive to SARs interventions, and which may find them less suitable.

Compared to other digital intervention platforms such as tablets or smartphones, SARs offer distinct advantages in promoting engagement and adherence in mental health interventions. Their embodied, human-like form enables multimodal communication, combining speech, gestures, and facial expressions, which fosters a stronger sense of social presence and emotional connection with users (3, 5, 6). Previous studies have shown that embodied agents can enhance motivation and therapeutic alliance more effectively than screen-based devices, particularly among individuals with affective or cognitive impairments (8–10).

While tablet-based cognitive training provides valuable accessibility and familiarity, it remains limited to two-dimensional interaction and lacks reciprocal social feedback. In contrast, humanoid SARs can actively perceive and respond to users’ emotions, deliver verbal encouragement, and adapt their behavior in real time. These features make them particularly suitable for psychiatric rehabilitation, where emotional engagement and motivational support are critical for therapy adherence (11, 12).

The usability and acceptance of SARs are essential factors for their successful integration into clinical settings (13). Attitudes towards robots are influenced by cultural, demographic, and individual factors (14). High usability is crucial for both patients and healthcare providers because it facilitates effective use and encourages acceptance (15). This is especially important for patients with depression, where cognitive impairments and emotional factors may pose additional challenges to usability (16). Acceptance refers to patients’ willingness to interact with the SAR and incorporate it into their treatment, whereas usability describes the robot’s user-friendliness and effectiveness in practical applications (3). These aspects are particularly significant in healthcare, where perceived usefulness, user-friendliness, and trust in technology play pivotal roles (13). Research has consistently shown that the overall usability and acceptance of robots in healthcare are positive (5, 17–20). The usability and acceptance of SARs vary among different patient groups. For example, Pu et al. (21) found that older adults generally have a positive attitude towards SARs, particularly when these robots are perceived as supportive rather than replacements for human interaction. In psychiatric care, Huijnen et al. (22) observed that patients with autism spectrum disorders benefit from interacting with SARs, indicating the potential usability and acceptance of SARs within this group. Broadbent et al. (3) emphasized that aspects of user-friendliness, such as intuitive interaction design, adaptability to user needs, and smooth navigation with minimal technical errors, are critical determinants of acceptance and willingness to engage with healthcare robots among older adults. This underscores the importance of designing intuitive and accessible human-robot interactions to ensure the successful adoption of SAR in diverse clinical settings.

Furthermore, baseline clinical factors such as depression severity may influence patients’ engagement with digital or robot-assisted interventions. Higher symptom severity has been associated with reduced motivation, concentration, and psychomotor activity, which can limit active participation and perceived usability (23, 24). Likewise, pharmacological treatment may affect cognitive processing speed or emotional responsiveness, potentially moderating interaction quality and engagement. Considering these aspects is essential for understanding variability in patient responses to SAR-supported interventions (25).

Sex-specific differences in the acceptance and use of technology are also important aspects that must be considered in SAR research, particularly in psychiatric settings. Previous studies suggest that men and women may exhibit different preferences and attitudes towards robots, with some findings indicating higher acceptance of caregiving robots among women (26–28). These differences may significantly influence the perception and use of robots in clinical contexts. Despite the growing interest in SAR in healthcare, there is a lack of studies focusing on the usability and acceptance of such technologies among psychiatric inpatients with depression. Existing research has primarily examined older adults, individuals with dementia, and general medical populations (13, 14). Thus, there remains a need to explore how psychiatric patients, particularly those with depression, experience and evaluate SAR, while also accounting for sex-specific perspectives to inform the development of more tailored and sensitive robotic interventions (26).

This study aims to address recent research gaps by investigating the usability and acceptance of the SAR “Pepper” when used as an adjunct motivational technology in combination with tablet-based cognitive training, compared with tablet-based training alone without motivational feedback, among inpatients with depression in psychiatric care. Specifically, this study investigated how inpatients with depression in psychiatric care evaluate the usability and acceptance of a SAR-enhanced intervention, while additionally examining sex-specific differences in patients’ evaluation of the intervention. For this purpose, we hypothesized that patients with depression who complete cognitive training sessions using the SAR “Pepper” with motivational feedback would report higher 1) usability and 2) acceptance than those receiving the same training via tablet, with significant sex-specific differences in their evaluations.

## 2 Methods and materials

### 2.1 Design, setting and sample

This pilot study was designed and conducted as a randomized controlled parallel two-arm trial (RCT) following the recommendations of the CONSORT 2025 Statement (29) to ensure transparency and methodological rigor in reporting. The RCT was carried out between June and October 2024 at the Clinical Division of Psychiatry and Psychotherapeutic Medicine at the Medical University of Graz, Austria. The sample consisted of 32 inpatients with depression. The diagnosis of depression was made by the treating clinicians according to the ICD-10 criteria. To evaluate the severity of the current depressive symptomatology the Montgomery-Åsberg Depression Rating Scale (MADRS) (30, 31) and the Beck Depression Inventory – Revised (BDI-II) (32) were used. The MADRS is an observer-rated assessment tool that evaluates depressive symptoms over the past week. It consists of ten items rated on a 7-point Likert scale, with total scores ranging from 0 to 60. Scores between 0 and 6 indicate no depression, whereas scores above 34 suggest severe depression (30, 31). In contrast, the BDI-II is a self-report questionnaire in which patients assess their feelings during the past week. It comprises 21 items rated on a 4-point Likert scale, yielding a maximum score of 64 points. A score of 0–8 points indicates no signs of depression, 9–13 points suggests minimal depression, 14–19 points indicates mild depression, 20–28 points indicates moderate depression, and scores of 29 or higher indicate severe depression (32). All assessments were conducted by trained staff members in a designated quiet room that was specifically prepared for this purpose. Both tests were used solely to describe the basic characteristics of the sample and were not considered as outcome variables.

### 2.2 Sample size

The sample size calculation was originally based on a repeated-measures ANCOVA design with two measurement points (*η²* = 0.3, power = .90, *α* = .05), yielding a target of N = 32 participants (33). This approach was chosen because the broader project “AMIGA” (34, 35) included multiple pre–post measures, which are not part of the current publication. Although the present analysis focuses on post-intervention questionnaire data (SUS, TAM, TUI), the same total sample size was retained, as it meets the requirements for parametric testing based on the central limit theorem, which is generally satisfied for sample sizes of approximately 30 or more. “AMIGA” was conceptualized as a pilot study intended to generate initial evidence on usability and acceptance of SAR-supported cognitive training in depression. Consequently, the findings should be interpreted as exploratory and hypothesis-generating rather than confirmatory. The calculation was conducted using G*Power software (version 3.1.9.6) (36, 37).

### 2.3 Inclusion and exclusion criteria

The inclusion and exclusion criteria for the participants were as follows. Participants eligible for inclusion were men and women aged ≥ 18 years who met the ICD-10 criteria for moderate or severe depression. They had to be able to speak and understand German, give written informed consent, and had no physical limitations that would prevent the use of the SAR “Pepper”.

The exclusion criteria were refusal to participate, presence of psychotic disorders, organic brain diseases or dementia, substance-induced disorders, or acute suicidality.

### 2.4 Randomization and blinding

After the participants agreed to enroll, allocation to the intervention or control group was carried out through randomization using an online randomizer (https://www.studyrandomizer.com) (38), ensuring balanced randomization with respect to 1) sex, 2) depression severity according to the ICD-10 diagnosis at admission, and 3) age. Blinding was not performed in this study because the interventions were apparent and could not be concealed.

### 2.5 Study course

**Figure 1:**
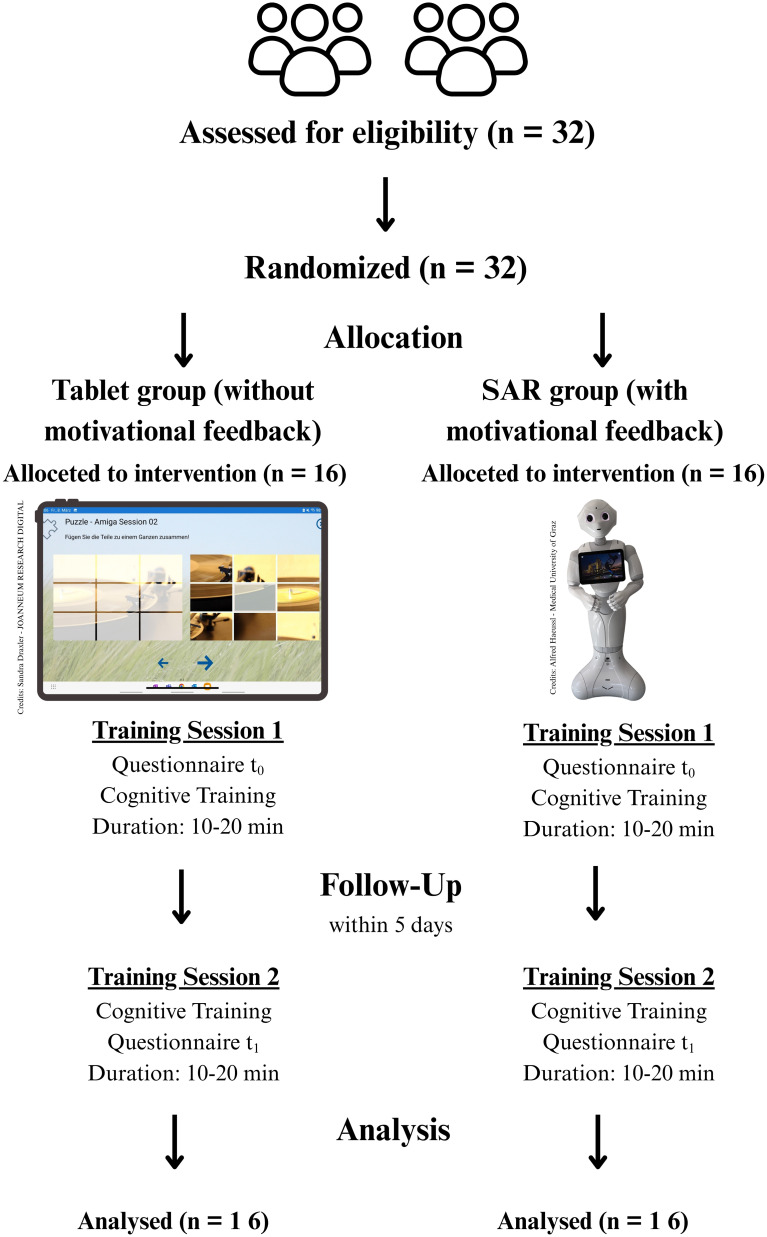
Study course and patient flow diagram according to Hopewell et al. (29).

**Figure 2:**
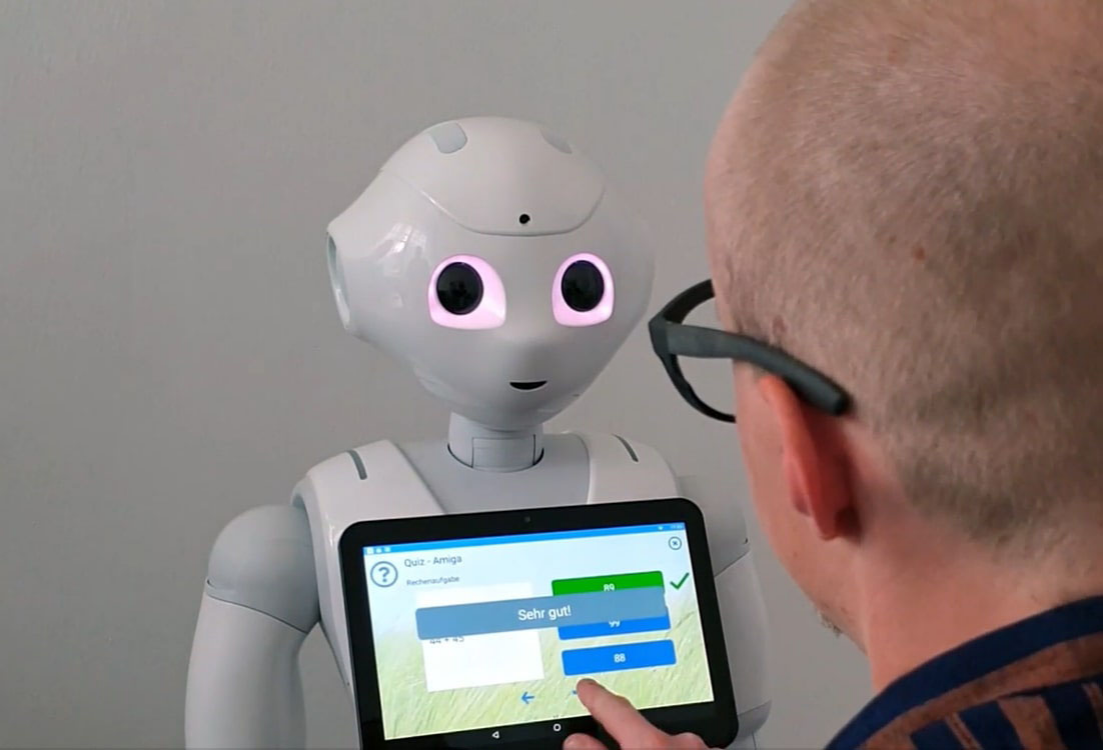
Participant interacts with the tablet-supported MMA application on the SAR “Pepper” and completes the cognitive task (solving arithmetic problems) (The participant provided written informed consent for this photograph to be taken and for its publication in the article; Credits: Sandra Draxler – JOANNEUM RESEARCH DIGITAL).

First, the participants were assigned to either an intervention group (SAR “Pepper” with motivational feedback) or a control group (tablet without motivational feedback). Over five days, participants in both groups had the opportunity to engage in cognitive training using the “Multimodal Activation” (MMA) application twice. In the intervention group, SAR “Pepper” was used to accompany and monitor the participants during training. After the participants completed the three tasks in cognitive training, SAR “Pepper” provided motivational feedback to encourage their progress. In contrast, the control group received the same cognitive training exercises, but these were conducted exclusively using the “MMA” app on an Android tablet (see **Figures 1**, **2**). Apart from this difference, the procedures were identical in both groups. The primary distinctions between the intervention and control conditions were the mode of delivery (SAR “Pepper” versus tablet) and the presence or absence of motivational feedback during training (no motivational feedback in the tablet group).

The first training session began with the participants completing the baseline questionnaires (t_0_) via LimeSurvey (https://www.limesurvey.org) (39). They then participated in the first cognitive training session. In the second session, they again completed a cognitive training session and filled out the post-intervention questionnaires (t_1_) using LimeSurvey. Subsequently, the participants completed the study. Each cognitive training lasted approximately 10 to 20 minutes.

### 2.6 SAR “Pepper”

SAR “Pepper” is a 120 cm tall and 28 kg heavy humanoid robot. SAR “Pepper” is capable of moving its arms, hands, and head and can analyze and respond to human emotions through body posture, speech, facial expressions, and voice tone. Equipped with microphones, SAR “Pepper” can identify and turn towards the person speaking (40–42). SAR “Pepper” fosters friendly and effective human-robot interaction to support therapy, rehabilitation, or learning, often enhancing motivation and quality of life (43).

### 2.7 “MMA”-app and cognitive training

The “MMA” (m_ultim_odal a_ctivation) application (19, 44) consists of a tablet-based front end and a back end installed on a central server. Both components were developed using Microsoft’s “.NET Framework”. The front end was created using Xamarin, a cross-platform framework by Microsoft that enables mobile application development in “.NET”. The application was specifically designed for Android and optimized for tablet displays. Additionally, an application programming interface was implemented to facilitate user management and data exchange, including authentication, version updates, and the transmission of exercise results. A structured query language database was developed to store the user data and the received results. Furthermore, a content management system was implemented, allowing the creation and categorization of various types of exercises into thematic units (45, 46).

Cognitive training consisted of three different exercise blocks, each comprising six tasks. Consequently, a total of 18 tasks were completed per training session. The difficulty level was adjusted after the completion of each exercise block and progressively increased with each step. The first exercise block was conducted at difficulty level 2 out of 4, the second block at difficulty level 3 out of 4, and the third block at the highest difficulty level, 4 out of 4.

Each training session included only three different cognitive tasks selected from distinct cognitive domains. In the first training session, participants were required to complete tasks such as finding picture pairs (domain: memory), solving arithmetic problems (domain: working memory), and filling in missing words in a text (domain: semantic memory). In the second training session, the tasks consisted of solving arithmetic problems (domain: working memory), completing puzzles (domain: visuospatial memory), and organizing a sequence of events in the correct order (domain: seriality and long-term memory).

The selection of cognitive tasks was based on established domains known to be affected in cognitive training, including memory, attention, executive functioning, and verbal fluency. Tasks were derived from the validated MMA training framework, which has previously been used in studies with neurological and psychiatric populations (19, 44–46). The difficulty levels were designed to progressively increase across sessions to sustain motivation and allow participants to experience a sense of achievement, while avoiding cognitive overload. This adaptive structure follows cognitive rehabilitation principles suggesting that gradual task escalation enhances engagement and supports learning in individuals with cognitive and affective impairments (47).

### 2.8 Questionnaires

#### 2.8.1 System usability scale

The System Usability Scale (SUS) is a questionnaire designed to evaluate technology usability. The advantage of the SUS lies in its technology-independent nature and brevity, making it quick and easy to complete. It consists of 10 questions rated on a five-point Likert scale (0 = strongly disagree to 4 = strongly agree). The items (2, 4, 6, 8, 10) need to be reverse-scored before further calculations, as they are negatively worded. Subsequently, the sum of all 10 items was calculated and multiplied by 2.5. This step yielded a total score (SUS score). A total score of 68 or higher indicates good usability (48–50). In the literature, the SUS is described as a reliable scale (ω = .89 and Cronbach’s alpha α = .86) that effectively detects differences compared to other commercially available questionnaires, even with small sample sizes (51). Its validity, in this case, perceived user-friendliness, was also rated highly (52).

#### 2.8.2 Technology usage inventory

The Technology Usage Inventory (TUI) evaluates the psychological and technology-specific factors that influence technology adoption. The TUI instrument consists of 30 items distributed across eight subscales: Curiosity, Anxiety, Interest, User-Friendliness, Usefulness, Scepticism, and Accessibility. The *Immersion* subscale, although part of the standard TUI, was intentionally excluded from this study, as immersion was not applicable to either the robot or tablet condition. This modification aligns with the TUI manual guidelines. Each item was rated on a seven-point Likert scale (1 = “Strongly Disagree” to 7 = “Strongly Agree”). Subscales with three items (Accessibility and User-Friendliness) yielded scores ranging from 3 to 21 points, while subscales with four items (Anxiety, Curiosity, Interest, Usefulness, and Scepticism) ranged from 4 to 28 points, resulting in a total possible TUI score between 26 and 182 points. Additionally, the Intention to Use scale, comprising three items, was included, allowing participants to rate their intentions on a scale from 0 to 100 per item, for a total score ranging from 0 to 300. The TUI has demonstrated robust psychometric properties, with internal consistencies (Cronbach’s alpha) ranging from.70 to.89 (53).

#### 2.8.3 Technology acceptance model

The Technology Acceptance Model (TAM) by Davis (54) represents a central approach in acceptance research and serves as a foundation for many subsequent models (55). This theory aims to explain the individual usage behavior of new technologies and their influencing factors, with the goal of predicting technology use. This model is based on two significant influencing factors: ‘perceived usefulness’ and ‘perceived ease of use’ of the technology. Perceived usefulness refers to the extent to which individuals believe that using a particular system enhances their performance (54). Therefore, a high level of perceived usefulness is believed to positively influence users’ performance. Perceived ease of use describes the extent to which individuals assume that using a system requires little effort (54). Individuals evaluate a technology with a high perceived ease of use as simple to operate (54). Each scale consisted of six questions. The two subscales Perceived Usefulness and Perceived Ease of Use demonstrated excellent internal consistency, with Cronbach’s alphas of.98 and.94, respectively (54). Additionally, two further questions were included to assess the intention to use the app. The questionnaire consisted of 14 items, which were answered using a seven-point Likert scale (1 = “Strongly Disagree” to 7 = “Strongly agree”).

### 2.9 Analysis

All statistical analyses were performed using IBM SPSS Statistics software (version 30) (56). Demographic characteristics were analyzed using descriptive statistics, including means (*M*) and standard deviations *(SD)*. The usability and acceptance scales were evaluated exclusively at the second measurement point (t_1_). To examine group and sex differences in the questionnaire outcomes, different statistical approaches were applied based on the structure of the respective instruments. For questionnaires consisting of a single overall scale (i.e., SUS and the overall score of the TUI), a one-way analysis of co-variance (ANCOVA) was performed. For instruments comprising multiple subscales (i.e., TAM and the subscales of the TUI), a multivariate analysis of co-variance (MANCOVA) was conducted. Statistical significance was set at *p* <.05 (two-tailed hypothesis testing) for all analyses. Prior to conducting ANCOVA and MANCOVA, all statistical assumptions were tested and met. To account for potential baseline imbalances between group and sex, ANCOVAs and MANCOVAs were adjusted for baseline depression severity (BDI-II t_0_ and MADRS t_0_ scores). Adjusted effect sizes (partial *η²*) and 95% confidence intervals (*CI*s) are reported. Bonferroni corrections were applied to control for multiple comparisons in both the ANCOVA and MANCOVA analyses.

## 3 Results

### 3.1 Characteristics of the participants

The study included 32 inpatients with moderate to high depression symptom severity according to ICD-10 criteria. The mean age of participants was 38.12 years (*SD* = 14.03; range = 19–65), with an equal distribution of women (*n* = 16) and men (*n* = 16) across the SAR group (with motivational feedback) and tablet group (without motivational feedback). Overall, the participants presented with moderate depressive symptomatology, as indicated by mean MADRS scores of 30.63 (± 4.99) and BDI-II scores of 25.44 (± 8.57). The groups were comparable in age and sex composition, while the SAR group (with motivational feedback) showed slightly higher baseline depression severity than the tablet group (without motivational feedback) (see **Table 1**). No participants dropped out during the study period, and all completed both training sessions and questionnaires. Data were checked for completeness before analysis; no cases with missing values were identified. Therefore, all 32 participants were included in the final analyses. Mean scores (*M*), standard deviations and (*SD*) and 95% confidence intervals of the SUS, the TUI and the TAM are presented in **Table 2**.

**Table 1:** Demographic and clinical characteristics of the sample.

Variable	SAR group (with motivational feedback)	Tablet group (without motivational feedback)	Total
Female (*n*)	8	8	16
Male (*n*)	8	8	16
Age *(M, SD)*	37.69 (14.59)	38.56 (13.91)	38.12 (14.03)
MADRS *(M, SD)*	32.06 (4.37)	29.19 (5.28)	30.63 (4.99)
BDI-II *(M, SD)*	28.25 (9.61)	22.63 (6.51)	25.44 (8.57)

BDI-II, Beck Depression Inventory; M, Mean; MADRS, Montgomery Åsberg Depression Rating Scale; SAR, Socially Assistive Robot; SD, Standard Deviation.

**Table 2:** Descriptive statistics of system usability scale, technology usage inventory and technology acceptance modell between SAR and tablet group and sex.

Variables	SAR group (with motivational feedback) (*n* = 16)				Tablet group (without motivational feedback) (*n* = 16)			
	*M*	*SD*	95% CI		*M*	*SD*	95% CI	
			*Lower bound*	*Upper bound*			*Lower bound*	*Upper bound*
SUS	77.50	10.165	72.08	82.93	61.72	9.904	56.44	67.00
TUI OVERALL	98.94	13.051	91.98	105.89	102.19	16.710	93.28	111.09
TUI INT	19.25	4.973	16.60	21.90	16.06	5.221	13.28	18.84
TUI USE	16.00	7.659	11.92	20.08	17.88	4.500	15.48	20.27
TUI SCE	14.13	4.689	11.63	16.62	10.13	4.530	7.71	12.54
TUI ANX	13.25	4.669	10.76	15.74	9.88	5.772	6.80	12.95
TUI UF	17.63	3.594	15.71	19.54	14.56	3.098	12.91	16.21
TUI ACC	7.56	3.054	5.93	9.19	10.31	4.029	8.17	12.46
TUI CUR	18.50	5.514	15.56	21.44	16.00	5.797	12.91	19.09
TUI ITU	206.38	37.137	186.59	226.16	185.00	77.562	143.67	226.33
TAM PU	30.56	3.949	28.46	32.67	32.63	3.914	30.54	34.71
TAM PEU	34.19	3.582	32.28	36.10	30.88	4.544	28.45	33.30
TAM ITU	11.88	1.586	11.03	12.72	11.00	1.592	10.15	11.85
Variables	Female (*n* = 16)				Male (*n* = 16)			
	*M*	*SD*	95% CI		*M*	*SD*	95% CI	
			*Lower bound*	*Upper bound*			*Lower bound*	*Upper bound*
SUS	69.69	13.130	62.69	76.68	69.53	12.722	62.75	76.31
TUI OVERALL	106.38	16.272	97,70	115.05	94.75	10.878	88.95	100.55
TUI INT	17.56	5.278	14.75	20.38	17.75	5.434	14.85	20.65
TUI USE	18.31	6.750	14.72	21.91	15.56	5.585	12.59	18.54
TUI SCE	13.69	5.056	10.99	16.38	10.56	4.501	8.16	12.96
TUI ANX	12.06	5.531	9.12	15.01	11.06	5.482	8.14	13.98
TUI UF	16.63	3.500	14.76	18.49	15.56	3.829	13.52	17.60
TUI ACC	9.44	4.589	6.99	11.88	8.44	2.828	6.93	9.94
TUI CUR	18.69	5.724	15.64	21.74	15.81	5.492	12.89	18.89
TUI ITU	210.94	56.600	180.78	241.10	180.44	62.762	146.99	213.88
TAM PU	32.81	4.151	30.60	35.02	30.38	3.575	28.47	32.28
TAM PEU	33.25	4.583	30.81	35.69	31.81	4.151	29.60	34.02
TAM ITU	11.69	1.401	10.94	12.43	11.19	1.834	10.21	12.16

*M* and *SD* are used to represent mean and standard deviation including the 95% confidence interval for each item.

ACC, Accessibility; ANX, Anxiety; CI, confidence interval; CUR, Curiosity; INT, Interest; ITU, Intention to Use; M, Mean; PEU, Perceived Ease of Use; PU, Perceived Usefulness; SAR, Socially Assistive Robot; SCE, Scepticism; SD, Standard deviation; Sig, Significance; SUS, System Usability Scale; TAM, Technology Acceptance Model; TUI, Technology Usage Inventory; UF, User-Friendliness; USE, Usefulness.

### 3.2 System usability scale

Bonferroni-corrected *post-hoc* analysis revealed a significant difference between SUS scores of the SAR group (with motivational feedback) and the tablet group (without motivational feedback (*p* <.001, *M*_Diff_ = 15.317, 95%-CI[6.938, 23.696]), after adjusting for baseline depression scores (BDI-II t_0_ and MADRS t_0_). No statistically significant results were found for sex comparisons (see **Table 3**).

**Table 3:** Comparison of system usability scale and technology usage inventory overall between SAR and tablet group and sex after adjusting for baseline depression scores (BDI-II t_0_ and MADRS t_0_).

Dependent variable	(I)	(J)	Mean difference (I-J)	*F*	Sig. ^a^	*η²_p_*	95% CI for difference ^a^	
							Lower bound	Upper bound
SUS	SAR ^b^	Tablet ^c^	15.317	14.022	<.001***	.334	6.938	23.696
TUI OVERALL	SAR ^b^	Tablet ^c^	-4.635	.582	.452	.020	-17.085	7.815
SUS	Female	Male	-.104	-001	.982	.000	-9.411	9.204
TUI OVERALL	Female	Male	11.479	5.011	.033*	.152	.974	21.983

ANCOVA. *N* = 32.

η²p, Partial Eta Squared; SAR, Socially Assistive Robot; Sig, Significance; SUS, System Usability Scale; TUI, Technology Usage Inventory.

* p <.05. ** p <.01. *** p <.001.

a Adjustment for multiple comparisons: Bonferroni.

b with motivational feedback.

c without motivational feedback.

The values shown in bold indicate statistically significant results. Depending on the level of significance achieved, they are marked with *, **, or ***.

### 3.3 Technology usage inventory

Bonferroni-corrected *post-hoc* analysis revealed a significant difference between TUI overall scores of females and males (*p* = .033, *M*_Diff_ = 11.479, 95%-CI [.974, 21.983]), after adjusting for baseline Depression scores (BDI-II t_0_ and MADRS t_0_) (see **Table 3**).

After adjusting for baseline depression scores (BDI-II t_0_ and MADRS t_0_), a multivariate analysis of covariance (MANCOVA) revealed significant group differences for two subscales of the TUI.

Participants in the SAR group (with motivational feedback) reported significantly higher scores in the *User-Friendliness* subscale compared to the Tablet group (without motivational feedback), *F (1*, 28) = 12.18, *p* = .002, η²_p_ = .303, 95% CI [1.81, 6.95]. In contrast, the tablet group showed significantly higher scores in the subscale *Accessibility* than the SAR group, *F (1*, 28) = 8.07, *p* = .008, η²_p_ = .224, 95% CI [–6.70, –1.09].

A further significant effect was found for sex in the subscale *Scepticism*, with female participants reporting higher scores than male participants, *F*(1, 28) = 4.34, *p* = .046, η²_p_ = .134, 95% CI [0.06, 6.90].

### 3.4 Technology acceptance model

After adjusting for baseline depression scores (BDI-II t_0_ and MADRS t_0_), a multivariate analysis of covariance (MANCOVA) revealed no statistically significant results for all subscale for group and sex comparisons (see **Table 4**).

**Table 4:** Comparison of the technology usage inventory and the technology acceptance model between SAR and tablet group and sex after adjusting for baseline depression scores (BDI-II t_0_ and MADRS t_0_).

Dependent variable	(I)	(J)	Mean difference (I-J)	*F*	Sig. ^a^	*η²_p_*	95% CI for difference ^a^	
							Lower bound	Upper bound
TUI INT	SAR ^b^	Tablet ^c^	3.744	3.315	.079	.106	-.472	8.019
TUI USE	SAR ^b^	Tablet ^c^	-1.997	.604	.444	.021	-7.264	3.269
TUI SCE	SAR ^b^	Tablet ^c^	3.583	3.651	.066	.115	-.258	7.424
TUI ANX	SAR ^b^	Tablet ^c^	2.944	2.150	.154	.071	-1.169	7.056
TUI UF	SAR ^b^	Tablet ^c^	4.381	12.175	.002**	.303	1.809	6.953
TUI ACC	SAR ^b^	Tablet ^c^	-3.893	8.069	.008**	.224	-6.698	-1.085
TUI CUR	SAR ^b^	Tablet ^c^	3.701	2.683	.113	.087	-.927	8.330
TUI ITU	SAR ^b^	Tablet ^c^	34.995	2.315	.139	.076	-12.121	82.111
TAM PU	SAR ^b^	Tablet ^c^	-2.906	3.522	.071	.112	-82.111	12.121
TAM PEU	SAR ^b^	Tablet ^c^	2.568	2.512	.124	.082	-.751	5.888
TAM ITU	SAR ^b^	Tablet ^c^	.803	1.547	.224	.052	-.520	2.126
TUI INT	Female	Male	-.143	.005	.943	.000	-4186	3.900
TUI USE	Female	Male	2.714	1.413	.245	.048	-1.963	7.391
TUI SCE	Female	Male	3.480	4.340	.046*	.134	.058	6.902
TUI ANX	Female	Male	1.283	.475	.496	.017	-2.528	5.093
TUI UF	Female	Male	.959	.510	.481	.018	-1.791	3.708
TUI ACC	Female	Male	1.110	.643	.429	.022	-1.726	3.946
TUI CUR	Female	Male	2.792	1.830	.187	.061	-1.435	7.019
TUI ITU	Female	Male	29.271	1.974	.171	.066	-13.399	71.941
TAM PU	Female	Male	2.549	3.320	.079	.106	-.317	5.414
TAM PEU	Female	Male	1.808	1.483	.233	.050	-1.233	4.849
TAM ITU	Female	Male	.570	.941	.340	.033	-.633	1.774

MANCOVA. *N* = 32.

ACC, Accessibility; ANX, Anxiety; CUR, Curiosity; INT, Interest; ITU, Intention to Use. η²p, Partial Eta Squared; PEU, Perceived Ease of Use; PU, Perceived Usefulness; SAR, Socially Assistive Robot; Sig, Significance; SCE, Scepticism; TAM, Technology Acceptance Model; TUI, Technology Usage Inventory; UF, User-Friendliness; USE, Usefulness.

*p <.05. **p <.01. ***p <.001.

a Adjustment for multiple comparisons: Bonferroni.

b with motivational feedback.

c without motivational feedback.

The values shown in bold indicate statistically significant results. Depending on the level of significance achieved, they are marked with *, **, or ***.

## 4 Discussion

This study aimed to evaluate the usability and acceptance of the SAR “Pepper” when implemented as a motivational adjunct to tablet-based cognitive training, compared to cognitive training delivered without robotic support, in inpatients with depression, with a particular focus on sex-specific differences. These findings provide valuable insights into the integration of robotic technologies into psychiatric care. Given the increasing interest in technology-based interventions for mental health care, understanding user preferences and technological acceptance is crucial for the successful implementation of such interventions (8).

Regarding technology usability and acceptance, the results revealed several significant findings. Usability ratings measured via the SUS and user-friendliness measured via TUI User-Friendliness favored the SAR group (with motivational feedback), with significantly higher scores compared to the tablet group (without motivational feedback). In contrast the tablet group (with motivational feedback) showed significantly higher scores in the TUI subscale Accessibility. Additionally, sex-related differences were observed, with female participants demonstrating significantly higher TUI Overall and Scepticism subscale scores than males.

These findings highlight that while the robot-assisted intervention was perceived as usable and user-friendly, it was also met with higher skepticism. This finding indicates that higher usability does not necessarily translate into greater overall acceptance. The SAR condition appears to evoke ambivalent reactions, as participants appreciated the interactive and social features while simultaneously expressing reservations regarding the SAR interaction.

SAR “Pepper’s” usability was also rated positively, indicating a user-friendly design for human-robot interaction for patients with depression. These results align with previous research, indicating that SARs tend to be rated as highly engaging due to their interactive nature and human-like characteristics (8, 57). For example, the robot ‘Ryan’, which delivers internet-based cognitive behavioral therapy, has been shown to improve mood scores and engagement in older adults with depression (9). This underscores the importance of intuitive usability for the successful integration of robotic technology into clinical environments. Similar findings were reported by (11), who demonstrated that high usability improved SAR acceptance and utilization among older adults. Notably, patients with depression, who often face cognitive impairments (16), reported no significant difficulties in operating the robot. The user-friendly approach of SAR “Pepper” may be attributed to its perception as a supportive complement to traditional therapy rather than a replacement for human interaction (5). This interpretation is supported by Göransson et al. (58), who found that patients evaluated SARs more positively when they were presented as an addition rather than a substitute for human contact. Broadbent (3) emphasized the importance of intuitive and user-friendly design in overcoming barriers to technology use and facilitating its integration into clinical settings.

Qualitative feedback collected informally after the sessions supported these findings: several participants in the SAR group described SAR “Pepper” as “interesting but somewhat unusual at first,” and some expressed uncertainty about “how natural” the interaction felt compared with human contact. Others reported technical aspects such as delayed speech recognition or limited gesture responsiveness as minor obstacles to immersion. Similar concerns have been described in previous research, where users reported feelings of unfamiliarity, artificiality, or reduced authenticity in early stages of human–robot interaction (59, 60).

Furthermore, these findings align with those of Bishop et al. (17), who documented a generally positive attitude towards healthcare robots. Da Rocha Ferreira et al. (5) also highlighted the importance of clear communication regarding the added value of SAR in driving their acceptance. The results also showed that interacting with “Pepper” was perceived as enjoyable and motivating. This is particularly important because motivation and engagement play central roles in treating depression. The positive perception of usability underscores the importance of well-thought-out human-robot interactions to foster the acceptance and practical use of SAR.

Despite these advantages, skepticism toward SARs remains a notable barrier to acceptance. In our study, females reported significantly higher skepticism scores than men. This finding aligns with research suggesting that while SARs may be initially engaging, long-term usability and trust issues may arise, particularly among individuals with higher levels of anxiety or resistance to new technologies (61). Similar concerns were raised in studies evaluating the robot ‘Paro’, where some participants expressed reluctance to engage with a robotic intervention, perceiving it as artificial or intrusive (10, 62).

The significantly higher accessibility scores reported by participants in the tablet group (without motivational feedback) compared to those in the SAR group (with motivational feedback) can be attributed to a combination of cost- and usability-related factors. Tablets are generally more affordable and readily available, making them more accessible for a broader user base (63, 64). In contrast, SARs involve high upfront costs because of their complex design, restricting their use primarily to high-income settings (65). From a usability perspective, tablets benefit from familiar touchscreen interfaces and intuitive navigation, although certain design aspects, such as small screen size or complex applications, can potentially hinder their effectiveness for users with physical or cognitive limitations (66). Although SARs are specifically designed for ease of use, their technical limitations, including the need for structured training, may negatively impact perceived accessibility among older or mentally ill users (67). Overall, these findings underscore that the tablet’s combination of economic feasibility and relative usability likely contributed to its higher accessibility ratings in the current study.

A central aspect of this study was the exploration of sex-specific differences in the usability and acceptance of SAR “Pepper”. The results revealed that women tended to have a higher overall usability of SARs than men. This observation is consistent with the findings of Kuo et al. (26), who examined age- and sex-specific differences in the perception and acceptance of healthcare robots. Moradbekhti et al. (27) also found that women rated SARs more positively, particularly regarding its usefulness and user-friendliness. The reasons for these sex-specific differences are complex. Ringwald et al. (28) argue that such differences may be attributed to distinct socialization experiences and role expectations. Women, often characterized by stronger caregiving orientations and social sensitivity, may be more receptive to using supportive technologies. Additionally, they may place greater emphasis on the emotional and social aspects of interacting with SARs than men. This suggests that the relationship between sex and SAR acceptance is complex and potentially context-dependent. The sex-specific findings of this study highlight the necessity of incorporating sex-sensitive approaches into the development and implementation of SARs. This could involve targeted adjustments in human-robot interaction or differentiated communication strategies that address the specific needs and expectations of different sexes.

Another critical aspect of SARs adoption is the role of sex-specific preferences. Recent research suggests that men and women may have different expectations of SARs, with women placing greater emphasis on emotional support and social interaction and men prioritizing functional aspects and usability (68). This may explain some of the variability in user engagement and skepticism observed in the present study. Furthermore, studies have highlighted that the personality adaptation of SARs can significantly influence user acceptance. For example, SARs with extroverted personalities tend to be more accepted by extroverted users, whereas introverted users may prefer calmer, less expressive robotic interactions (69, 70). These findings emphasize the importance of adaptive, user-tailored robot interactions, a concept that has been incorporated into SAR interventions, such as this project (35).

A recurring challenge in SAR-based interventions is the sustainability of engagement over time. While our study found a preference for the SAR over the tablet in terms of usability and user friendliness, it remains unclear whether this preference would persist over an extended period. Previous research indicates that SAR engagement often decreases after an initial novelty phase, leading to reduced long-term adherence (10, 62). A possible explanation for this phenomenon is the limited adaptability of SARs, which may lead users to perceive interactions as repetitive or lacking meaningful engagement over time (12).

Additionally, ethical concerns regarding privacy, data security, and autonomy have been raised in previous studies, particularly in relation to psychiatric patients (61). While SARs offer promising solutions for cognitive training and emotional support, their successful implementation in clinical practice requires addressing the above-mentioned barriers to trust and acceptance.

The positive results regarding the usability and acceptance of SAR “Pepper” among patients with depression highlight the promising opportunities for SARs use in psychiatric care. SARs can serve as a complementary tool in depression treatment, assisting with cognitive training exercises, providing information, and offering emotional support. Studies such as those by Chen et al. (71) have demonstrated that SAR-based intervention programs can positively affect cognitive function and mood in older adults in long-term care facilities. Similarly, Chen et al. (72) reported successful SAR interventions in children with autism spectrum disorders. These findings suggest that SARs could be versatile tools for treating depression.

## 5 Strengths and limitations

This study provides valuable insights into the usability and acceptance of SARs and tablet-based cognitive training in individuals with depression. A key strength of this study is the randomized group assignment, which minimizes selection bias and ensures comparability between the intervention groups. By using validated psychometric instruments, such as the MADRS and BDI-II, this study offers robust clinical assessments of depressive symptoms, increasing the reliability of the findings.

Another strength of this study is the use of multiple standardized measures to assess technology usability and acceptance. The inclusion of the SUS, the TUI, and the TAM allows for a comprehensive evaluation of the user experience.

Additionally, this study contributes to the growing body of research on SARs in mental health care by examining their clinical usability. Previous research has mainly focused on older adults or individuals with cognitive impairments (9, 57), whereas this study specifically addressed depression in an adult population, thereby expanding the applicability of SAR interventions to broader psychiatric settings.

Despite its promising findings and strengths, this study has several limitations that should be acknowledged. The main limitation concerns the asymmetry in feedback between the two groups. The SAR group included verbal motivational feedback of the SAR “Pepper”, whereas the tablet condition did not provide any motivational feedback beyond task progression. This introduces an additional factor, that may have contributed to the higher usability and acceptance ratings in the SAR group. Consequently, the results should be interpreted as reflecting the combined effect of humanoid interaction and motivational feedback rather than the robotic embodiment alone. Future studies should equalize feedback across conditions to isolate the specific contribution of the SAR component.

The second limitations was the relatively small the sample size (*n* = 32), which may limit the generalizability of the findings. While the randomized design strengthens internal validity, the statistical power of the study remains restricted, particularly in detecting subtle effects or interaction effects between the variables. Future studies should aim to replicate these findings in larger and more diverse samples to enhance their external validity.

Another limitation relates to the limited intervention duration and training frequency of the cognitive training intervention. Participants completed only two training sessions, which may not have been sufficient to produce measurable improvements in cognitive performance. Prior studies have indicated that long-term engagement is crucial for the sustained benefits of SAR-based interventions, as engagement levels may decline after an initial novelty phase (10, 62). Abdi et al. (73) emphasized the importance of long-term studies to better understand the lasting impact and acceptance of SARs in healthcare settings. Future research should explore the effects of prolonged SAR interventions on cognitive and emotional outcomes.

The sex-specific differences observed in this study warrant further investigation in future studies. Intersectional aspects should also be considered to develop a nuanced understanding of the factors influencing SAR usability and its acceptance. Eyssel and Hegel (74) emphasized the need to incorporate cultural and socioeconomic factors into the analysis to obtain a holistic perspective on this issue.

This study focused exclusively on patients with depression. Future research should explore the use of SARs for other psychiatric conditions, such as anxiety disorders, bipolar disorders, and post-traumatic stress disorders. Studies by Huijnen et al. (22) and Rabbitt et al. (75) have shown promising results for the use of SARs among patients with autism spectrum disorders and other psychiatric diseases.

Beyond the methodological and conceptual limitations discussed above, two additional aspects warrant further attention. The first relates to the integration of SARs into existing treatment frameworks. While this study focused on the evaluation of usability and acceptance, it did not examine how SAR-based cognitive training interacts with standard therapeutic approaches. Therefore, future studies should investigate whether combining SAR interventions with conventional treatments can enhance therapeutic outcomes. As highlighted by Tapus et al. (76), such integrative approaches are crucial for harnessing the full potential of SARs in psychiatric care.

Another important consideration is the ethical dimensions of SAR use in sensitive clinical environments. Although not the focus of this study, issues such as data privacy, informed consent, and unintended psychosocial consequences of human-robot interactions are highly relevant, particularly when working with vulnerable populations. Addressing these questions in future research is essential to ensure ethical implementation. Sharkey and Sharkey (77) emphasize the importance of proactively engaging with ethical challenges in the context of healthcare robotics.

## 6 Conclusion

The overall positive evaluation of SAR “Pepper” underscores the potential of SARs as a complementary tool in psychiatric care. The observed sex-specific differences highlight the need for tailored approaches to developing and implementing SAR technology in clinical environments.

The results of this study provide a solid foundation for further research and advancement of SAR technology in psychiatric care. They highlight the potential of SAR technology to enhance treatment quality and open new pathways for patient care. Simultaneously, they caution against an overreliance on SAR and stress the need to view them as complements, not replacements, to human interaction and traditional therapies.

Future research should investigate the long-term effects of SAR in psychiatric care, extend its applicability to other mental health conditions, and explore complex interactions between SAR technology and traditional treatment approaches. Only through continuous research and careful evaluation can we fully harness the potential of SAR in psychiatric care while ensuring their use remains ethical and patient centered.

## Data Availability

The original contributions presented in the study are included in the article/supplementary material. Further inquiries can be directed to the corresponding author.
